# Cognitive Dysfunction and Learning Implications in Medical Students With Depressive Symptoms: Electrophysiological Evidence From P300 Event-Related Potentials

**DOI:** 10.7759/cureus.99806

**Published:** 2025-12-21

**Authors:** Ricardo Jesús Martínez-Tapia, Arantza Martínez-Zarraluqui, Diana Guízar-Sánchez, Raúl Sampieri-Cabrera

**Affiliations:** 1 Department of Physiology, School of Medicine, National Autonomous University of Mexico, Mexico City, MEX; 2 Department of Medicine, Anahuac University, Mexico City, MEX; 3 Center for Complexity Sciences, National Autonomous University of Mexico, Mexico City, MEX

**Keywords:** cognitive dysfunction, depression, event-related potentials, medical students, neuropsychology, p300

## Abstract

Introduction: Major depressive disorder is highly prevalent among medical students and strongly associated with cognitive dysfunctions.

Objective: To compare neuropsychological and electrophysiological profiles (P300 parameters) of medical students with and without depressive symptoms.

Methods: A cross-sectional and comparative study was conducted with 140 second-year medical students. Depressive symptoms were assessed with the Patient Health Questionnaire-9. Cognitive performance was evaluated with the Montreal Cognitive Assessment (MoCA) and CogniFit (CogniFit Inc., San Francisco, CA, USA) computerized tests. Event-related potentials were recorded through a standard auditory oddball paradigm, analyzing N100, N200, and P300 latency and amplitude. Statistical analyses included independent sample t-tests and analysis of variance, with significance set at p < 0.05. Effect sizes (Cohen’s d) were reported for all group comparisons, and appropriate corrections for multiple comparisons were applied to control type I error.

Results: Students with depressive symptoms exhibited slower response time (p < 0.01), processing speed (p = 0.01), impaired contextual memory (p =.01), short-term memory (p = 0.01), working memory (p = 0.01), focused attention (p = 0.01) and perception domain( p = 0.03). On the MoCA, lower in abstraction (p = 0.03), delayed memory recall (p < 0.01), and total MoCA score (p = 0.008). Event-related potentials analysis revealed significantly prolonged latencies for N100, N200, and P300 (all, p < 0.01), and decreased amplitudes in N100, N200, and P300 (p < 0.05). Prolonged event-related potentials latencies (particularly P300) correlated negatively with performance on processing speed (r = -0.39, p < 0.001), focused attention (r = -0.32, p < 0.001), and delayed recall (r = -0.31, p < 0.001).

Conclusions: Medical students with depressive symptoms demonstrate specific cognitive impairments and altered event-related potential markers, reflecting reduced attentional efficiency and information processing. Combining computerized neuropsychological testing with electrophysiological measures may provide a feasible screening pathway for cognitive vulnerability among medical trainees and guide the development of preventive and educational interventions within medical curricula.

## Introduction

Major depressive disorder (MDD) is a leading cause of disability worldwide, affecting nearly 5% of the global adult population [1]. Beyond affective and somatic symptoms, cognitive dysfunction has emerged as a central feature of depression, with deficits in executive functions, attention, working memory, and processing speed not being secondary, but rather core components that significantly impair functional recovery and quality of life [2-5]. Estimates suggest that 85-90% of patients experience cognitive impairment during acute depressive episodes, and nearly 40% continue to exhibit deficits even after clinical remission [6,7].

Cognitive dysfunction, defined as impairments in core mental processes such as attention, memory, executive functioning, and information processing, has critical implications for education and occupational performance [3,8]. Reduced processing speed, impaired executive functioning, and compromised memory may hinder academic achievement, occupational performance, and interpersonal relationships [8]. Persistent cognitive deficits are also strong predictors of relapse, poor treatment outcomes, and reduced psychosocial adjustment [9]. Therefore, the early identification of cognitive deficits in individuals with depressive symptoms is critical for improving functional prognosis.

Depression is a major public health challenge in the university context. The prevalence of depressive symptoms among higher-education students ranges from 25% to 35%, exceeding those reported in the general population [10,11]. Medical students face heightened vulnerability owing to long training hours, academic overload, constant evaluations, and exposure to stressful clinical environments. These conditions not only increase the risk of depressive disorders but also exacerbate cognitive difficulties, compromising both academic performance and professional development [12]. Previous studies have reported that depression in medical students is associated with higher rates of burnout, suicidal ideation, and academic dropout [10,13,14]. However, despite growing attention to student mental health, few studies have systematically examined how depressive symptoms interact with cognitive and neurophysiological processes in non-clinical young populations, such as medical students [15,16]. This limited evidence stands in contrast to the abundance of event-related potential (ERP)-based studies in clinical MDD samples, highlighting the need for early, subclinical biomarkers in educational contexts [17-21].

Despite the well-documented burden of depression among medical trainees, most investigations have focused on affective and somatic symptoms, with limited attention paid to cognitive and neurophysiological correlations [10,22]. While standardized neuropsychological tests provide valuable information about specific domains [4,7], they rely on subjective performance and are influenced by contextual variables, such as motivation or fatigue. In this regard, electrophysiological techniques offer an opportunity to obtain objective markers of cognitive function [17,19,20]. 

ERPs are time-locked electrophysiological responses derived from scalp EEG that reflect synchronous excitatory and inhibitory postsynaptic potentials generated by cortical pyramidal neurons [17,19,20]. They provide a non-invasive, inexpensive, and temporally precise method for examining neurocognitive processes relevant to psychopathology [17,20]. ERPs are commonly elicited using the auditory oddball paradigm, in which a continuous train of frequent auditory stimuli is intermittently interrupted by infrequent “rare” stimuli that differ in intensity or acoustic features [19]. These rare stimuli were presented in a randomized order to prevent predictability, maintain sustained attention, and avoid contingency effects associated with regular stimulus intervals. Importantly, P300 responses can also be elicited when a rare event consists of the omission of an expected frequent stimulus, underscoring the paradigm’s versatility in probing attentional and cognitive processing.

ERPs waveforms comprise multiple components indexing the distinct stages of information processing. Early components include the N100 (N1), which emerges around 100 ms and reflects initial sensory encoding and early selective attention, and the N200 (N2), which appears around 180-300 ms and is associated with executive control mechanisms such as stimulus evaluation, conflict monitoring, novelty detection, inhibitory control, and mismatch processing [23]. P300 (P3), a positive deflection peaking between 250-400 ms, is the most widely studied late component in depression research and reflects attentional allocation, working-memory updating, and context integration. It consists of two subcomponents: P3a, an earlier frontocentral peak occurring 250-300 ms and ~20-50 ms before P3b, which is maximal over parietal regions and peaks around 300-350 ms [17,19,20]. Quantitatively, the P300 amplitude represents the voltage difference (in μV) between the pre-stimulus baseline and the largest positive peak, indexing the neural resources devoted to stimulus processing, whereas latency reflects the time (in ms) to the peak and indicates processing speed [17,19,20].

The P300 component reflects neuroelectric responses to attention and working memory demands. Prolonged latency has been associated with slower cognitive processing, where reduced amplitude indicates a diminished allocation of attentional resources [24,25]. Evidence suggests that P300 alterations may be sensitive biomarkers of cognitive dysfunction in MDD [26-28].

However, data integrating both cognitive testing and ERP measures in medical students with depressive symptoms remains scarce [29]. At the time of the study, we did not find any studies that systematically compared neuropsychological profiles with electrophysiological parameters, despite their high relevance for early detection and preventive interventions. Such integration could provide novel insights into the mechanisms through which depressive symptoms could be one of the factors associated with academic performance, as well as inform tailored strategies to mitigate the long-term consequences of cognitive dysfunction within medical training.

Therefore, this study aimed to compare neuropsychological performance and electrophysiological parameters (specifically, the P300 component) between medical students with and without depressive symptoms. By combining standardized cognitive assessment with objective neurophysiological markers, this study sought to contribute novel evidence on the cognitive inefficiencies and attentional processing alterations associated with depressive symptoms in young, non-clinical populations, offering potential implications for early detection and preventive educational strategies.

## Materials and methods

Study design and participants

This cross-sectional, comparative study was conducted between October 2022 and April 2023 among undergraduate medical students enrolled in the second year of a six-year medical program at a Mexican public university. Participants were recruited through institutional announcements and selected via non-probabilistic convenience sampling, an approach commonly used in educational and mental-health research because of logistical and feasibility constraints in university settings.

The inclusion criteria were (a) enrollment as a full-time medical student; (b) age between 18 and 25 years; and (c) right-handedness to ensure EEG consistency. Exclusion criteria included (a) history of neurological or psychiatric disorders (other than depressive symptoms screened in this study); (b) use of psychoactive medication, substances, or consumption of more than 200-300 mg of caffeine (approximately ≥2 cups of coffee) within 24 hours prior to testing; (c) sleep deprivation (<6 hours of sleep the night prior); and (d) uncorrected visual or auditory impairments.

Participants were classified into two groups, with and without depressive symptoms, based on validated cut-off scores from the Patient Health Questionnaire-9 (PHQ-9), where a score of ≥10 indicated clinically relevant depressive symptomatology [30,31]. The PHQ-9 was also used to determine symptom severity, following the established classifications: 0-4 (minimal), 5-9 (mild), 10-14 (moderate), 15-19 (moderately severe), and 20-27 (severe). The instrument demonstrated strong psychometric performance, with internal consistency coefficients (Cronbach’s α) of 0.86-0.89 and sensitivity and specificity values of approximately 0.88, supporting its validity for identifying depressive symptoms. All classifications were determined prior to cognitive and electrophysiological testing to avoid measurement bias [30,31].

Neuropsychological assessment

Cognitive performance was assessed using two complementary tools, the CogniFit (CogniFit Inc., San Francisco, CA, USA) Cognitive Assessment Battery (CAB) and Montreal Cognitive Assessment (MoCA).

CogniFit Cognitive Assessment Battery (CAB)

The CogniFit Cognitive Assessment Battery (CAB), is a validated computerized neuropsychological battery that evaluates more than 20 cognitive abilities across domains, including memory (short-term, working, contextual, and recognition), attention (focused, divided, and sustained), executive functions (planning, inhibition, updating, and flexibility), visuospatial perception, reaction time, and processing speed. The full assessment takes approximately 30-40 minutes. Raw scores were normalized for age and sex using standardized normative data, providing domain-specific indices. CogniFit has shown acceptable psychometric properties. In recent validation work, internal consistency (Cronbach’s α = 0.85-0.88) and test-retest reliability (r = 0.69-0.92) have been reported [32]. The battery is widely used in research and by health professionals to screen for cognitive strengths and weaknesses in diverse populations [33-35].

Montreal Cognitive Assessment (MoCA)

The Montreal Cognitive Assessment (MoCA) is a standardized screening instrument for global cognition covering attention, memory, executive functions, language, visuospatial abilities, and orientation, with total scores ranging from 0 to 30 points. Higher scores reflect better cognitive functioning, and a validated Spanish version was used [36]. A cutoff score of ≤25 was used to indicate possible cognitive impairment. This threshold is consistent with findings from the Mexican validation study by Aguilar-Navarro et al., which reported optimal sensitivity and specificity for detecting mild cognitive impairment using scores ≤26 in older adults, independent of age and education [36]. Given our younger, highly educated sample, the ≤25 cutoff provides an appropriate and conservative criterion aligned with the validated performance characteristics of the MoCA.

This dual assessment was selected to integrate the ecological validity of the MoCA with the domain precision of CogniFit, thus enabling a multidimensional understanding of cognitive efficiency and executive performance.

Electrophysiological assessment (event-related potential)

ERPs were recorded using a 32-channel electroencephalography (EEG) system, Cadwell EEG/EP system (Cadwell Industries, Kennewick, WA, USA) with a sampling rate of 1000 Hz, and electrode impedance was maintained below 5 kΩ, following the international 10-20 system. All recordings were conducted in an electrically and acoustically shielded room under dim lighting, with the participants seated comfortably. The procedure was explained to all the participants. Then, with all the necessary precautions, the electrodes were positioned on the scalp at positions Fz, Cz, and Pz, with linked mastoids as the reference and Fpz as the ground. Vertical and horizontal electrooculogram (EOG) channels were used to monitor eye movements.

The EEG data were first cleaned using a band-pass filter of 0.1-30 Hz to remove slow drifts and high-frequency noise. The continuous EEG was then divided into time segments (epochs) ranging from 100 ms before the stimulus to 800 ms after it. Each epoch was adjusted using a −100 to 0 ms baseline period to ensure that all segments started at a comparable voltage level. To reduce artifacts, ocular activity (blinks and eye movements) was identified and removed using independent component analysis (ICA). Any remaining epochs with excessive noise (greater than ±100 µV) were automatically excluded. After preprocessing, an average of 82-90% of all trials were retained, and every participant contributed at least 30 artifact-free target trials, providing a reliable signal-to-noise ratio for ERP analysis.

A standard auditory oddball paradigm was used, with 80% frequent tones (1000 Hz) and 20% infrequent target tones (1500 Hz) with a 1000 ms interstimulus interval. The participants were instructed to press a button when detecting the target tones. Stimuli were delivered in a pseudorandom sequence to prevent predictability, and the participant had to be attentive to the task at hand. This paradigm is widely accepted as the preferred procedure for eliciting the P300 component [19,20].

Analyses focused on the N100, N200, and P300 components, measured at the central (Cz) and parietal (Pz) electrodes. The peak latency (ms) and amplitude (µV) were extracted using semi-automatic detection windows (N100: 60-120 ms, N200: 150-250 ms, P300: 280-500 ms).

Special attention was given to the P300, which is typically elicited between 300 and 600 ms after infrequent stimuli, reflecting selective attention, stimulus evaluation, and working memory updating. Alterations in these parameters have been associated with cognitive dysfunction in psychiatric disorders, including major depression.

All cognitive and electrophysiological assessments were performed at the Learning and Mental Health Sciences Laboratory under controlled lighting, temperature, and noise conditions to minimize external interference with signal quality and cognitive performance. The Committee on Ethics and Research, National Autonomous University of Mexico, approved the study protocol FM/DI/056/2021, and all participants provided written informed consent prior to enrollment. The study was conducted in accordance with the Declaration of Helsinki and adhered to international ethical principles for research involving human subjects.

Statistical analysis

Data normality was confirmed using Shapiro-Wilk tests, and homogeneity of variances was confirmed using Levene’s test. Independent-sample t-tests and one-way ANOVA were used to compare neuropsychological and ERP parameters between groups. Effect sizes are reported as Cohen’s d with 95% confidence intervals (CIs).

Correlation analyses between cognitive performance and ERP measures (latency and amplitude) were conducted using Pearson’s r, controlling for age and sex. Statistical significance was set at p < .05 (two-tailed), with false discovery rate (FDR) correction applied to control for multiple comparisons. All analyses were conducted using IBM SPSS Statistics (version 24.0; IBM Corp., Armonk, NY, USA).

To evaluate robustness, post-hoc power analyses (G*Power 3.1, Heinrich-Heine-Universität Düsseldorf, Düsseldorf, Germany) indicated that the achieved sample size (n = 140) provided >0.90 power to detect medium effect sizes (d = 0.50) at α = 0.05.

## Results

A total of 140 second-year medical students participated in the study (mean age = 21.0 ± 1.6 years), of whom 60.7% (n = 85) were female subjects. The majority were single (97.1%) and lived with their family (96.4%). No significant differences were observed between groups with and without depressive symptoms regarding age, sex, or socioeconomic status (all p > 0.05), ensuring comparability across groups.

Among participants with depressive symptoms, 74.3% (n = 52) scored in the moderate-to-severe range, and 25.7% (n = 18) were classified as having mild depression (Table 1).

**Table 1:** Sociodemographic and clinical characteristics of the sample. *χ² = chi-square test for categorical variables; **t = Student’s t-test for continuous variables.

Variable	Total n (%)	Without depressive symptoms n (%)	With depressive symptoms n (%)	Statistics
Total	140	70	70	–
Sex				
Women	85 (60.7)	47 (61.4)	38 (54.2)	p = 0.12*
Men	55 (39.3)	23 (38.6)	32 (45.8)	
Age (years, M ± SD)	21.6 ± 1.06	21.6 ± 1.01	21.7± 1.11	p = 0.50**
Relationship status				
Without partner	136 (97.1)	67 (95.7)	69 (98.5)	p = 0.31*
With partner	4 (2.9)	3 (4.3)	1 (1.5)	
Living arrangement				
With family	135 (96.4)	68 (97.1)	67 (95.7)	p = 0.64*
Without family	5 (3.6)	2 (2.9)	3 (4.3)	
Financial resources				
Sufficient	101 (72.1)	50 (71.4)	51 (72.8)	p = 0.85*
Insufficient	39 (27.9)	20 (28.6)	19 (27.2)	
Degree of depression				
None	70 (50)	70 (100)	0 (0)	–
Mild	0 (0)	0 (0)	0 (0)	
Moderate	27 (19.6)	0 (0)	27 (38.6)	
Moderately severe	25 (17.9)	0 (0)	25 (35.7)	
Severe	18 (12.5)	0 (0)	18 (25.7)	

Electrophysiological correlates (ERPs)

Table 2 presents a comparison of the ERP components between students with and without depressive symptoms. Students with depressive symptoms exhibited systematic alterations in the ERP components. Latency measures were significantly prolonged for N100 (M = 95.5 ± 8.9 ms) compared to controls (M = 78.4 ± 8.5 ms; t = 11.63, p < 0.001, d = 2.0), for N200 (M = 198.7 ± 14.0 ms vs. 169.9 ± 14.8 ms; t = 11.77, p < 0.01, d = 2.0), and for P300 (M = 309.6 ± 13.8 ms vs. 280.5 ± 15.7 ms; t = 11.63, p < 0.01, d = 2.0).

**Table 2:** ERP latency and amplitude measures between students with and without depressive symptoms. Latency values are expressed in milliseconds (ms), and amplitude values are expressed in microvolts (µV). Student’s t-test was used for between-group comparisons. ERP = electrophysiological correlate.

ERP component	Measure	Without depressive symptoms (M ± SD)	With depressive symptoms (M ± SD)	t	p-value	Cohen's d
N100	Latency (ms)	78.4 ± 8.5	95.5 ± 8.9	11.63	<0.001	2.0
	Amplitude (µV)	-2.95 ± 1.09	-2.23 ± 0.84	-4.37	<0.001	0.7
N200	Latency (ms)	169.9 ± 14.8	198.7 ± 14.0	11.77	<0.01	2.0
	Amplitude (µV)	-4.45 ± 1.50	-3.36 ± 1.06	-4.93	<0.001	0.8
P300	Latency (ms)	280.5 ± 15.7	309.6 ± 13.8	11.63	<0.01	2.0
	Amplitude (µV)	9.59 ± 3.59	8.17 ± 3.24	2.44	0.016	0.4

Amplitude values were also reduced in students with depressive symptoms for N100 (-2.23 ± 0.84 µV vs. -2.95 ± 1.09 µV; t = -4.37, p < 0.001, d = 0.7), N200 (-3.36 ± 1.06 µV vs. -4.45 ± 1.50 µV; t = -4.93, p < 0.001, d = 0.8), and P300 (8.17 ± 3.24 µV vs. 9.59 ± 3.59 µV; t = 2.44, p = 0.016, d = 0.4).

**Figure 1:**
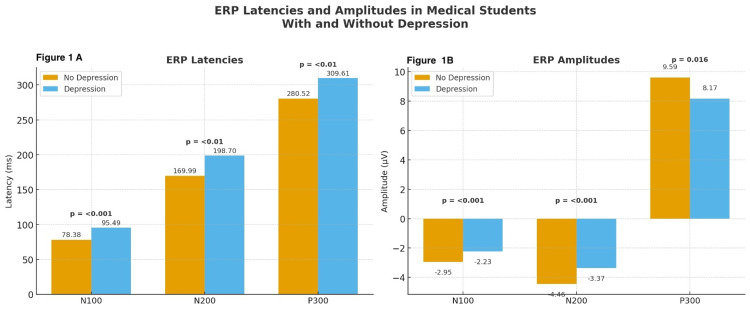
Latency and amplitude of N100, N200, and P300 components in medical students with and without depressive symptoms. (1A) Group differences in ERP latencies for N100, N200, and P300 components. Values represent mean latencies (ms) with corresponding p-values derived from independent-sample t-tests. Students with depressive symptoms showed a significantly prolonged latency across all components. (1B) Group differences in ERP amplitudes for N100, N200, and P300 components. Bars depict mean amplitudes (µV); p-values were obtained using independent-samples t-tests. Students with depressive symptoms demonstrated significantly reduced amplitudes across all ERP components. ERP = electrophysiological correlate.

These findings demonstrated a consistent pattern of slowed neural processing and reduced allocation of attentional resources among students with depressive symptoms. The large effect sizes observed for latency variables suggest a robust neurophysiological divergence between groups. Figure 1 illustrates a comparison of ERPs between students with and without depressive symptoms, showing clear latency delays (Figure 1A) and amplitude reductions (Figure 1B) in students with depressive symptoms.

Global cognition (MoCA)

On the Montreal Cognitive Assessment (MoCA), no significant group differences were found in visuospatial/executive function, attention, language, or orientation (all p > 0.05). However, students with depressive symptoms scored significantly lower in abstraction (1.75 ± 0.44 vs. 1.88 ± 0.32; t(138) = 2.11, p =0.03, d = 0.4), delayed memory recall (2.67 ± 1.17 vs. 3.52 ± 1.28; t(138) = 4.13, p < 0.01, d = 0.7), and total MoCA score (22.75 ± 2.28 vs. 23.88 ± 2.69; t(138) = 2.68, p = 0.008, d = 0.5) (Table 3).

**Table 3:** Comparison of MoCA cognitive domains between students with and without depressive symptoms. Values represent the mean (M) ± standard deviation (SD). Higher scores indicated better cognitive performance. Interpretation of Cohen’s d: 0.20 = small, 0.50 = medium, 0.80 = large. MoCA: Montreal Cognitive Assessment

Cognitive domain	Without depressive symptoms	With depressive symptoms	Statistics	Cohen’s d
Visuospatial	3.76 (1.140)	3.70 (1.115)	t=0.340, p=0.734	0.05
Identification	2.99 (0.487)	2.93 (0.468)	t=-0.020, p=0.984	0.5
Attention	3.97 (1.483)	3.67 (1.390)	t=-1.256, p=0.211	0.15
Language	1.96 (0.836)	1.88 (0.850)	t=0.517, p=0.606	0.10
Abstraction	1.88 (0.323)	1.75 (0.438)	t=2.111, p= 0.03	0.35
Memory	3.52 (1.275)	2.67 (1.172)	t=4.126, p=<0.01	0.7
Orientation	5.94 (0.374)	5.97 (0.169)	t=-0.555, p=0.58	0.10
Total	23.88 (2.69)	22.75 (2.28)	t=2.683, p=0.008	0.45

Table 3 also displays the effect sizes (Cohen’s d) and statistical significance for each domain of the MoCA when comparing students with and without depressive symptoms. While most domains showed negligible or non-significant differences, students with depressive symptoms performed significantly worse in abstraction, memory, and total MoCA scores, with small to moderate effect sizes.

Specific cognitive processes (CogniFit)

Table 4 presents the comparative neuropsychological assessment using CogniFit, and significant differences were observed between the groups with and without depressive symptoms in multiple cognitive domains. Students with depression scored significantly lower in planning (68.31 ± 16.34 vs. 76.90 ± 15.04; p = 0.01, d = 0.6) and processing speed (66.86 ± 18.24 vs. 82.74 ± 11.16; p = 0.01, d = 1.0), reflecting a generalized slowing in the execution of cognitive tasks. They also performed worse on contextual memory (63.66 ± 21.24 vs. 74.94 ± 21.32; p = 0.01, d = 0.5), short-term memory (62.30 ± 19.72 vs. 74.39 ± 16.99; p = 0.01, d = 0.7), and working memory (62.67 ± 18.51 vs. 75.57 ± 17.83; p = 0.01, d = 0.7).

**Table 4:** Comparison of CogniFit cognitive domains between students with and without depressive symptoms. M = mean; SD = standard deviation. Comparisons based on independent-sample t-tests. Cohen’s d reflects the magnitude of effect size. Interpretation of Cohen's d: 0.20 = small, 0.50 = medium, 0.80 or greater = large.

Cognitive domain	Without depressive symptoms	With depressive symptoms	Statistics	Cohen's d
Planning	76.90 (15.04)	68.31 (16.34)	t=3.235 p=0.01	0.6
Processing speed	82.74 (11.16)	66.86 (18.24)	t=6.214 p=0.01	1
Cognitive flexibility	77.20 (18.57)	74.93 (17.00)	t=0.755 p=0.452	0.15
Short-term phonological memory	68.26 (22.13)	62.54 (21.23)	t=1.559 p=0.121	0.53
Contextual memory	74.94 (21.32)	63.66 (21.24)	t=3.137 p=0.01	0.5
Naming	71.06 (21.56)	68.59 (22.44)	t=0.664 p=0.508	0
Short-term memory	74.39 (16.99)	62.30 (19.72)	t=3.884 p=0.01	0.7
Nonverbal memory	66.96 (19.80)	68.99 (20.15)	t=-0.601 p=0.508	0.23
Short-term visual memory	69.80 (18.70)	65.27 (21.01)	t=1.347 p=0.180	0.30
Working memory	75.57 (17.83)	62.67 (18.51)	t=4.285 p=0.01	0.7
Divided attention	76.99 (15.78)	72.37 (15.33)	t=1.766 p=0.080	0.13
Focused attention	86.84 (11.52)	72.07 (14.45)	t=5.911 p=0.01	1.1
Inhibition	72.16 (15.13)	71.06 (19.69)	t=0.371 p=0.711	0.06
Monitoring	69.63 (21.72)	66.60 (23.32)	t=0.795 p=0.428	0
Eye-hand coordination	67.06 (19.63)	61.40 (22.38)	t=1.590 p=0.114	0.27
Response time	79.97 (12.94)	72.81 (14.53)	t=3.077 p=0.01	0.5
Auditory perception	70.99 (20.92)	65.64 (22.78)	t=1.446 p=0.151	-1.13
Estimation	62.80 (25.47)	56.79 (30.39)	t=1.269 p=0.207	0.04
Recognition	69.20 (23.78)	63.01 (22.82)	t=1.570 p=0.119	0.23
Spatial perception	61.29 (25.78)	53.99 (30.92)	t=1.517 p=0.132	0.27
Visual perception	73.40 (22.48)	64.73 (24.40)	t=2.187 p=0.030	0.4
Visual scanning	77.96 (17.22)	80.09 (18.60)	t=-0.703 p=0.483	0.40

In the attentional domain, significant differences were also identified in focused attention (72.07 ± 14.45 vs. 86.84 ± 11.52; p = .01, d = 1.1) and response time (72.81 ± 14.53 vs. 79.97 ± 12.94; p = 0.01, d = 0.5), suggesting alterations in the efficiency and sustainability of attentional processes. Finally, the visual perception domain showed a lower score in the group with depression (64.73 ± 24.40) than in the group without symptoms (73.40 ± 22.48; p = 0.03, d = 0.4). In contrast, other domains such as cognitive flexibility, nonverbal memory, inhibition, naming, and monitoring did not show statistically significant differences between the groups (Table 4).

Cognition-ERP correlation

Convergent evidence has emerged between cognitive and electrophysiological domains. Prolonged ERP latencies (particularly P300) were negatively correlated with performance in processing speed (r = -0.39, p < 0.001), focused attention (r = -0.32, p < 0.001), and delayed recall (r = -0.31, p < 0.001). Similarly, reduced P300 amplitude correlated positively with visuospatial and auditory perception (r ≈ 0.30, p < 0.001), suggesting that more efficient sensory integration is associated with stronger cortical responses, are summarized in Table 5. Notably, the correlation between processing speed and P300 latency was stronger in the control group (r = -0.46, p < 0.001) than in the depression group (r = -0.28, p = 0.015), indicating a decoupling between behavioral and neural efficiency among students with depressive symptoms. 

**Table 5:** Summary of significant ERP-cognition associations for P300 latency and amplitude. ERP =  event-related potential; r = Pearson correlation coefficient. All p-values are two-tailed.

ERP measure	Direction of association	Cognitive domain	Correlation (r)	p-value	Interpretation
P300 latency	Negative	Processing speed	−0.39	<0.001	Longer latencies associated with poorer processing speed.
	Negative	Focused attention	−0.32	<0.001	Delayed neural processing linked to reduced attentional efficiency.
	Negative	Delayed recall	−0.31	<0.001	Slower stimulus evaluation associated with poorer memory retrieval.
P300 amplitude	Positive	Visuospatial perception	0.278	<0.001	Greater amplitude reflects stronger perceptual integration.
	Positive	Auditory perception	0.305	<0.001	Higher cortical responsiveness linked to better auditory perceptual processing.

Table 6 summarizes the significant Pearson correlation coefficients between ERP components (N100, N200, P300) and performance across cognitive domains (e.g., MoCA subscales, processing speed, memory, attention, perception). Negative correlations indicate that longer ERP latencies or reduced amplitudes are associated with poorer cognitive functioning. Only statistically significant associations (p < 0.05) are shown.

**Table 6:** Correlations between ERP parameters and cognitive measures in students with and without depressive symptoms. Pearson correlation coefficients (r) and corresponding p-values are presented for participants with and without depressive symptoms. Negative correlations indicated lower cognitive performance associated with increased ERP amplitude or prolonged latency; positive correlations indicated better performance with greater cortical activation. Significant values are indicated in bold (p < 0.05). ERP = event-related potential; MoCA = Montreal Cognitive Assessment.

ERP measure	Cognitive domain	With depressive symptoms		Without depressive symptoms	
–	–	Pearson correlation coefficients (r)	p-value	Pearson correlation coefficients (r)	p-value
N100 amplitude	MoCA attention	-0.327	<0.001	0.394	< 0.001
	MoCA total score	-0.290	<0.001	0.379	< 0.001
	Processing speed	-0.208	0.014	0.117	0.886
	Divided attention	-0.200	0.018	0.208	0.082
	Response time	-0.196	0.020	0.112	0.351
	Focused attention	-0.191	0.024	0.131	0.067
	Estimation	0.167	0.049	0.305	0.01
N100 latency	Contextual memory	-0.326	<0.001	-0.161	0.181
	Processing speed	-0.295	<0.001	-0.003	0.784
	Hand-eye coordination	-0.291	<0.001	-0.019	0.873
	Focused attention	-0.287	<0.001	-0.104	0.388
	Working memory	-0.261	0.002	-0.186	0.121
	Visual perception	-0.260	0.002	-0.202	0.091
	Spatial perception	-0.247	0.003	-0.019	0.871
	Auditory perception	-0.237	0.005	-0.141	0.243
	Estimation	-0.227	0.007	-0.215	0.073
	MoCA delayed recall	-0.187	0.027	0.189	0.116
	Response time	-0.185	0.029	-0.046	0.070
	Recognition	-0.181	0.033	-0.061	0.610
	Naming	-0.167	0.048	-0.242	0.043
N200 amplitude	MoCA total score	-0.238	0.005	0.393	<0.001
	MoCA delayed recall	-0.230	0.006	0.215	0.073
	MoCA attention	-0.220	0.009	0.332	<0.001
	Response time	-0.180	0.034	0.132	0.034
	MoCA orientation	0.167	0.049	0.208	0.273
N200 latency	Focused attention	-0.337	<0.001	-0.035	0.769
	Contextual memory	-0.317	<0.001	-0.157	0.192
	Processing speed	-0.304	<0.001	-0.005	0.096
	Spatial perception	-0.254	0.002	-0.206	0.086
	Estimation	-0.222	0.009	-0.117	0.209
	Short-term phonological memory	-0.221	0.009	-0.186	0.121
	Hand-eye coordination	-0.181	0.032	-0.135	0.263
	MoCA abstraction	0.207	0.014	-0.088	0.467
P300 amplitude	MoCA visuospatial/executive	-0.272	0.001	-0.105	0.384
	MoCA total score	-0.228	0.007	0.241	0.044
	MoCA attention	-0.188	0.026	0.251	0.036
	MoCA language	-0.187	0.027	-0.041	0.735
	MoCA naming	0.169	0.045	0.188	0.118
	Nonverbal memory	0.173	0.041	0.157	0.193
	Cognitive flexibility	0.193	0.022	0.264	0.027
	Short-term memory	0.195	0.021	0.183	0.127
	Short-term phonological memory	0.197	0.020	0.139	0.250
	Recognition	0.203	0.016	0.299	0.097
	Naming	0.214	0.011	0.255	0.087
	Inhibition	0.218	0.010	0.238	0.047
	Working memory	0.262	0.002	0.315	0.007
	Visual perception	0.278	<0.001	0.309	0.009
	Auditory perception	0.305	<0.001	0.377	< 0.001
	Contextual memory	0.308	<0.001	0.245	0.041
	Hand-eye coordination	0.314	<0.001	0.253	0.034
	Estimation	0.351	<0.001	0.340	0.003
	Spatial perception	0.352	<0.001	0.285	0.016
P300 latency	Processing speed	-0.385	<0.001	-0.072	0.553
	MoCA delayed recall	-0.317	<0.001	-0.090	0.454
	Focused attention	-0.315	<0.001	-0.109	0.336
	Contextual memory	-0.313	<0.001	0.017	0.088
	Response time	-0.207	0.014	0.053	0.661
	Auditory perception	-0.196	0.020	0.151	0.209
	Recognition	-0.177	0.036	0.231	0.054
	Short-term phonological memory	-0.173	0.041	0.186	0.121
	Working memory	-0.173	0.041	0.069	0.566
	MoCA abstraction	0.214	0.011	0.088	0.467

Negative correlations between ERP measures and MoCA attention scores appeared in both the depressive-symptom and control groups, indicating a shared neurocognitive pattern in which greater cortical activation during attentional processing corresponds to lower performance. This suggests that an increased amplitude or prolonged latency reflects compensatory neural effort rather than enhanced efficiency. Importantly, these associations were consistently stronger in participants with depressive symptoms, indicating to a heightened vulnerability to neural inefficiency when attentional resources were challenged.

## Discussion

This study demonstrated that medical students with depressive symptoms exhibit specific cognitive impairments-particularly slowed information processing, reduced attentional efficiency, and executive dysfunction, as reflected in both behavioral performance and electrophysiological patterns. The combination of prolonged latencies and reduced amplitudes across ERP components (N100, N200, and P300) suggests disruptions in the neural mechanisms supporting attention and cognitive control. These findings reinforce previous evidence that depressive symptoms, even at subclinical levels, are associated with measurable inefficiencies in cognitive processing.

Neurophysiological correlates (ERPs)

The consistent prolongation of latencies and reduction in amplitudes across ERP components reflect slower neural processing and reduced allocation of attentional resources. Previous studies have shown that changes in P300 latency in MDD reflect less efficient information-processing strategies, which translate into slower attentional processing [27,37-39]. Furthermore, decreased P300 amplitude has been associated with impaired perception of environmental stimuli and allocation of cognitive resources [40]. The attenuated association between processing speed and P300 latency in students with depression suggests a decoupling of behavioral and neural efficiency, consistent with disrupted cognitive control mechanisms [41-43].

Integration with cognitive findings

The MoCA findings revealed modest decrements in abstraction and delayed recall, whereas CogniFit identified broader deficits in planning, working memory, and attentional control. These results align with previous evidence that depression preferentially affects higher-order processes [4,39]. The convergence between neuropsychological and electrophysiological data strengthens the interpretation that depression compromises complex reasoning, flexible attention, problem-solving abilities, and academic performance [8].

Academic and professional implications

Cognitive and electrophysiological alterations identified in medical students with depressive symptoms should be interpreted with caution. Although differences were observed in processing speed, executive function, and memory, the study did not assess academic performance or clinical competence; therefore, no direct implications for educational outcomes or future professional practice can be inferred. Instead, these results suggest the presence of neurocognitive patterns associated with depressive symptoms, which warrant further examination. Early identification through digital neuropsychological tools and electrophysiological markers such as the P300 may help characterize these patterns more precisely; however, these associations remain correlational, and their relevance to academic or functional outcomes can only be clarified through longitudinal research. Although approaches such as cognitive training, structured physical exercise, and neuromodulation-based interventions have shown promise in improving executive functioning [44], establishing their specific applicability to students with depressive symptoms requires prospective studies that directly assess changes over time. Likewise, although the P300 component has been proposed as a potential translational biomarker for monitoring treatment effects [41,42], its clinical utility remains contingent on systematic validation in well-designed longitudinal investigations.

This study had methodological strengths. First, the multimodal design (integrating self-report measures, neuropsychological performance, and electrophysiological markers) provided a more comprehensive characterization of the cognitive patterns associated with depressive symptoms in medical students. Second, all assessments were conducted under controlled laboratory conditions, which likely enhanced the reliability of both cognitive and ERPs recordings by minimizing environmental and measurement variabilities.

Limitations and future directions

This study has several limitations. The cross-sectional design prevents causal inference, and recruitment from a single university limits the generalizability of the findings. Moreover, the sample consisted exclusively of second-year medical students; therefore, the results may not be applicable to students in other stages of medical training or to those enrolled in dental or allied health programs. Future studies should include multiple academic years and a broader range of health professions to better capture developmental and curricular influences on cognitive and electrophysiological functioning. The cognitive alterations identified here, primarily involving processing speed, executive functions (planning and working memory), and memory, should be explored further using longitudinal and multicenter designs. Incorporating complementary methods, such as neuroimaging or multimodal biomarkers, may help clarify the evolution and functional significance of these cognitive and neural features in students with depressive symptoms.

## Conclusions

In the context of medical training, the cognitive and electrophysiological differences observed in students with depressive symptoms underscore the importance of identifying early signs of cognitive vulnerability. By integrating digital cognitive assessments with electrophysiological measures, this study offers a multidimensional perspective on how depressive symptoms relate to cognitive functioning among in medical students. Further research is needed to clarify how these cognitive and neural patterns evolve over time and determine their potential relevance to educational outcomes.
